# Impact Assessment of Mercury Accumulation and Biochemical and Molecular Response of *Mentha arvensis*: A Potential Hyperaccumulator Plant

**DOI:** 10.1155/2015/715217

**Published:** 2015-01-11

**Authors:** R. Manikandan, S. V. Sahi, P. Venkatachalam

**Affiliations:** ^1^Plant Genetic Engineering and Molecular Biotechnology Lab, Department of Biotechnology, Periyar University, Salem, Tamil Nadu 636 011, India; ^2^Department of Biology, Western Kentucky University, 1906 College Boulevard, No. 11080, Bowling Green, KY 42101-1080, USA

## Abstract

The present study was focused on examining the effect of Hg oxidative stress induced physiochemical and genetic changes in *M. arvensis* seedlings. The growth rate of Hg treated seedlings was decreased to 56.1% and 41.5% in roots and shoots, respectively, compared to the control. Accumulation of Hg level in both roots and shoots was increased with increasing the concentration of Hg. Superoxide dismutase (SOD), catalase (CAT), and ascorbate peroxidase (APX) activities were found to be increased with increasing the Hg concentration up to 20 mg/L; however, it was decreased at 25 mg/L Hg concentration. The POX enzyme activity was positively correlated with Hg dose. The changes occurring in the random amplification of ploymorphic DNA (RAPD) profiles generated from Hg treated seedlings included variations in band intensity, disappearance of bands, and appearance of new bands compared with the control seedlings. It was concluded that DNA polymorphisms observed with RAPD profile could be used as molecular marker for the evaluation of heavy metal induced genotoxic effects in plant species. The present results strongly suggested that *Mentha arvensis* could be used as a potential phytoremediator plant in mercury polluted environment.

## 1. Introduction

Mercury (Hg) heavy metal pollution is considered as a serious environmental problem throughout the world. Hg is a persistent neurotoxin and it is the only metal in the list of bioaccumulative chemicals of concern. Because of its chemical properties, it exists as an elemental metal in the form of mercuric ions and organomercury. Millions of tons of mercury has been released into the environment as a result of gold mining areas, industrial pollution, metal wastes, burning of fossil fuels, and electronics [1]. In the environment, Hg is converted by sulfate reducing bacteria to the extremely toxic compound methyl mercury which is bioaccumulated in the food chain [2, 3]. As heavy metals such as mercury do not decompose in the environment, effective strategies are needed to remove these compounds from the polluted sites. Environmental restoration of contaminated soils with traditional physical and chemical methods is quite expensive and environmentally invasive and demands extreme investments of economic and technological resources [4].

Heavy metals generally cause damage to plants, either directly or indirectly by triggering an increased level of production of reactive oxygen species (ROS). These ROS include superoxide radical (O_2_^•−^), hydroxyl radical (OH^•−^), and hydrogen peroxide (H_2_O_2_) that are produced as byproducts during membrane linked electron transport activities as well as by a number of metabolic pathways. ROS damage the cell membranes, nucleic acids, and chloroplast pigments [5]. Plants have antioxidant systems to protect them against oxidative damage. These detoxification processes are complex and highly compartmentalized in plant cells. The level of ROS in the plant is controlled by an antioxidative system that consists of antioxidative enzymes like SOD, CAT, APX, POX, and nonenzymatic low molecular mass antioxidants [6]. SOD is a major scavenger of superoxide anion free radical, which is converted into hydrogen peroxide (H_2_O_2_) and oxygen (O_2_) [7]. CAT that is localized in the peroxisomes scavenges H_2_O_2_ by converting it to H_2_O and O_2_. POD reduces H_2_O_2_ using several reductants of phenolic compounds [8]. Both APX and Glutathione S-transferase (GST) enzymes could play a pivotal role in scavenging ROS and maintaining the level of antioxidants ascorbate and glutathione [5]. Pb heavy metal toxicity inhibits chlorophyll synthesis by causing impaired uptake of essential elements such as Mg and Fe [9] and even accelerates the decomposition of chlorophyll [6].

Mercury heavy metal ion induces several cellular stress responses and damage to different cellular components such as membranes, proteins, and DNA. It binds strongly to a large number of molecules including DNA and RNA; it disrupts DNA synthesis and alters the transcriptional process and mitotic activity. Genome alterations consist of depolymerizations, generation of abnormal nitrogenous bases, DNA strand breaking, and DNA—DNA cross-links and DNA—protein cross-links. DNA damage may result in the production of abnormal bases [10]. Plants acting as biological indicators can measure the potential effect of pollutants when they are used to measure the effects of heavy metals. Recent advances in molecular biology have led to development of the number of selective and sensitive assays for DNA analysis in ecogenotoxicology. DNA fingerprinting techniques namely, RFLP, QTL, RAPD, AFLP, SSR, and VNTR, were used to detect the variation at the DNA base pairs level in the recent past. Random amplified polymorphic DNA (RAPD) analysis can be used to identify the differences in DNA fingerprints generated between control and heavy metal (genotoxic agents) treated individuals [11, 12]. RAPD is a reliable, sensitive, and reproducible assay that has the potential to detect a wide range of DNA damage, as well as mutation, and, therefore, it can be applied to study the genotoxicity [13, 14].

Phytoremediation is an emerging technology that can be applied for removal of heavy metal pollutants including Hg present in the soil and water bodies [2]. However the ability to accumulate heavy metals varies significantly between the plants species that have been identified as metal hyperaccumulators [15]. Hyperaccumulator plants are found in the families of Brassicaceae, Euphorbiaceae, Asteraccae, and Lamiaceae families [16]. The genus of* Mentha* belongs to the* Lamiaceae* family and has about 25–30 species [17].* Mentha* is one of the potential candidates for the phytoremediation of heavy metal contaminated soil. There are few reports on accumulation and tolerance to heavy metals cleanup by* Mentha aquatic *L.,* Mentha sylvestris *L., and* Mentha peppermint *[18, 19]. However, no report has been published on heavy metal accumulation/tolerance by* Mentha arvensis *species until now. In the present study, it is hypothesized that due to its multitolerance,* Mentha arvensis *had the potential to deal with heavy metal induced oxidative stress at the cellular level and we examined the detoxification capacity to cope with excess ROS generated by Hg heavy metal ions in the hydroponic solution. Therefore,* M. arvensis* seedlings were selected to study the Hg heavy metal hyperaccumulation potential and its oxidative stress induced physiochemical and genetic changes. The main objective of this study was to determine the Hg heavy metal accumulation level and to examine the effects of Hg exposure on biomass, photosynthetic pigments, total soluble protein contents, antioxidative enzyme (SOD, CAT, APX and POX) activities, and the level of DNA changes in* Mentha arvensis* seedlings.

## 2. Materials and Methods

### 2.1. Plant Growth Condition and Mercury Treatment

*M. arvensis* seedlings (20 days old) were collected from Horticultural Research Station, Tamil Nadu Agricultural University (TNAU), Yercaud, Tamil Nadu. The roots were washed several times in tap water to remove the soil particles for hydroponic experiments. Seedlings were transferred into plastic cups containing one letter of Hoagland nutrient solution (full strength) and provided proper aeration continuously for acclimatization. Subsequently, plants were treated with different concentrations of Hg (5, 10, 15, 20, and 25 mg/L), and Hg was supplied as mercury chloride (HgCl_2_) salt, while the medium without Hg served as control. After 12 days of treatment, seedlings were removed from the hydroponic solution and thoroughly rinsed with tap water and distilled water. The shoot and root tissues were collected separately, weighed, and used for determining mercury content level, antioxidative enzyme analysis, and genomic DNA isolation.

### 2.2. Determination of Mercury Content Level and Plant Growth Parameters

To determine the Hg content, shoot and root tissues were dried at 75°C for 48 h and then weighed separately. The root and shoot tissues were prepared for Hg quantification according to the method of Israr et al. [20]. The mercury content in root and shoot tissues was quantified using the method described by Liu et al. [21].

Both the shoot and root lengths were measured immediately after harvesting the seedlings and Index of tolerance (IT) for root and shoot was calculated according to Chen et al. [22]:

(1) $$\begin{array}{c} \text{IT}\left(\%\right)=\frac{\text{M}{\text{L}}_{\text{Hg}}}{\text{M}{\text{L}}_{\text{control}}}\times 100, \end{array}$$

where ML_Hg_ is the maximum root/shoots length of the seedlings in Hg with Hoagland solution divided by ML_control_ maximum root/shoot length of the seedlings in Hoagland solution without Hg.

To find out the relative water content, seedlings were harvested and the fresh weight (FW) of leaves was determined. The leaf samples were dried in a hot air oven for 48 hrs at 75°C for determination of dry weight (DW). The relative water content was estimated according to the equation of Chen et al. [22]:

(2) $$\begin{array}{c} \text{RWC}\left(\%\right)=\left[\left(\text{FW}-\frac{\text{DW}}{\text{FW}}\right)\right]\times 100, \end{array}$$

where RWC(%) is the relative water content, FW (g) is fresh weight of plants, and DW (g) is dry weight of seedlings. At the end of treatment,* M. arvensis* seedlings were divided into shoot and root. The fresh weight (FW) of the shoot and root were then measured. For DW estimation, the shoots and roots were dried at 65°C for 48 h.

### 2.3. Estimation of Photosynthetic Pigments

The photosynthetic pigments (chlorophyll *a*, *b* and Car) were determined according to the method of Arnon [23]. Briefly fresh leaves (100 mg) were homogenized in 80% (v/v) ice cold acetone and centrifuged at 5000 rpm for 5 min. The supernatant was collected and pellet was reextracted twice with 2 mL of 80% acetone. The absorbance of the supernatant was measured using Double beam UV-Visible spectrophotometer. The concentration of chlorophyll *a*, *b* and carotenoids was calculated using the following formula [24]:

(3) $$\begin{array}{c} \text{Chl}\;a=\frac{\left[\left(13.95A_{665}-6.88A_{649}\right)\times 10\right]}{100} \end{array}$$

(4) $$\begin{array}{c} \text{Chl}\;b=\frac{\left[\left(24.96A_{649}-7.32A_{665}\right)\times 10\right]}{100} \end{array}$$

(5) $$\begin{array}{c} \text{Car}=\left[\frac{\left(1000A_{470}-2.05\text{C}a-114.8\text{C}b\right)}{245}\right]\times \frac{10}{100}. \end{array}$$

### 2.4. Extraction and Assay of Antioxidative Enzymes (SOD, CAT, and POX) Activity

Fresh leaves mM phosphate buffer (pH 7.5) contains 0.5 mM EDTA. The homogenate was centrifuged or roots (0.2 g) were homogenized in a prechilled mortar pestle using under ice cold conditions with 2 mL of 50 at 10,000 rpm for 10 min. The supernatant was used as the crude extract for following antioxidative enzymes assay.

SOD activity was determined by the nitroblue tetrazolium (NBT) method as described by Dhindsa et al. [25]. The reaction mixture (3 mL) consisted of 50 mM phosphate buffer (pH 7.8), 13.33 mM methionine, 2.25 mM NBT, 0.1 mM EDTA, 50 mM NaCO_3_, 60 mM riboflavin, and enzyme extract. The absorbance was measured at 560 nm. One unit of SOD activity was defined as the amount of enzyme that produced 50% inhibition of NBT reduction under the assay conditions.

CAT activity was measured according to the method of Aebi [26]. The reaction mixture (3 mL) contained 50 mM phosphate buffer (pH 7.8), 75 mM H_2_O_2_, enzyme extract, and distilled water. The reaction was initiated by adding 75 mM of H_2_O_2_ and decrease in absorbance was recorded for 1 min at 240 nm using UV-Visible spectrophotometer.

APX activity was estimated according to the method of Nakano and Asada [27]. The reaction mixture (3 mL) contained 50 mM phosphate buffer (pH 7.8), 0.5 mM ascorbic acid 0.1 mM EDTA, 65 mM H_2_O_2_, enzyme extract, and distilled water. The oxidation of ascorbic acid was measured by the decrease in absorbance at 290 nm for 30 sec using UV-visible spectrophotometer (Double Beam Spectrophotometer 2203).

POX activity was measured using the method of Castillo et al. [28]. The reaction mixture (3 mL) contained 50 mM phosphate buffer (pH 6.1), guaiacol (16 mM), H_2_O_2_ (2 mM), enzyme, and distilled water. The oxidation of guaiacol was measured by the decrease in absorbance at 470 nm for 1 min using UV-visible spectrophotometer. Enzyme activities were expressed in Units per milligram fresh weight (U/mg fw).

### 2.5. Determination of Total Soluble Protein

Total soluble protein present in the supernatant was also determined according to Bardford [29] method using Bovine Serum Albumin (BSA) as standard and was expressed in mg/g fresh weight.

### 2.6. Genomic DNA Isolation

Total genomic DNA was extracted from the leaves by modified CTAB method [30]. Leaves (0.1 g) were homogenized in mortar and pestle with 1 mL of 2x CTAB buffer ((2% hexadecyl triethyl-ammonium bromide), 1.4 M NaCl, 20 mM EDTA (pH 8.0), 0.1 M Tris-HCl (pH 8.0), 1% polyvinyl polypyrolidone (PVPP), 1% (v/v) 2 mercaptoethanol) and incubated the extract in a water bath at 65°C for 30 min. It was spun at 8000 rpm for 10 minutes and the supernatant was transferred into new eppendrof tubes. This was reextracted with an equal volume of phenol : chloroform : isoamyl alcohol [25 : 24 : 1] and was centrifuged at 10,000 rpm for 10 min. The supernatant was collected into fresh tubes and RNAse (10 mg/mL) treatment was performed. The aqueous phase was reextracted with equal volume of chloroform and centrifuged at 10,000 rpm for 10 min. The supernatant was collected and 0.6 volume of ice cold isopropyl alcohol (100%) was added and the sample was kept on ice for 20 min. After centrifugation at 8,000 rpm for 10 minutes, the pellet was washed in 70% (v/v) ethanol. The pellet was air dried and dissolved in 1 mL of TE buffer and stored at −20°C for RAPD analysis.

### 2.7. PCR Amplification of Genomic DNA

Random decamer primers were purchased from Operon Technologies Inc., Alameda, CA, USA and were used for RAPD—PCR analysis [31]. The reaction was carried out in a volume of 20 *μ*L consisting of 1x PCR buffer (10 mM Tris-HCl) (pH 8.3): 50 mM KCl, 1.5 mM MgCl_2_, 1 mM dNTPs (dATP, dGTP, dCTP and dTTP), 0.5 unit of Taq DNA polymerase enzyme, and 25 ng of template DNA and 250 nM of RAPD primer and finally we added sterile water. Amplifications were performed in a thermal cycler under (Cyber Cycler-P series PCR peltier model p 96+ USA) the PCR amplification profile with first cycle at 94°C for 4 min, followed by 40 cycles at 94°C for 1 min/37°C for 1.5 min/72°C for 2 min with a final extension step at 72°C for 7 min. After completion of PCR cycles, loading dye was added to the amplified products and resolved by electrophoresis using 1.5% (w/v) agarose gels containing 0.5 mg/mL ethidium bromide in 1x TAE buffer. Electrophoresis was performed at 50 V power supply for 3 hrs, until the bromophenol blue front had migrated to the bottom of the gel. The molecular standard used was the lambda DNA double digested by* Eco*RI/*Hin*dIII. The gels were visualized and photographed under UV light using Alpha Innotech Gel Documentation system, USA. After screening, primers exhibiting clear banding pattern were selected for further RAPD analysis. The nucleotide sequences of the selected primers were provided in Table 3. Genomic template stability (GTS %) was calculated according to Liu et al. [21]:

(6) $$\begin{array}{c} \text{GTS}=\left(1-\frac{a}{n}\right)\times 100, \end{array}$$

where *a* is the number of polymorphic bands and *n* is the number of total bands in the control.

### 2.8. Statistical Analysis

For statistical validity, each treatment was made in 3 replicates for estimating enzyme activity and photosynthetic parameters and 3 replicates for root and shoot length measurement. The analysis of variance (ANOVA) was performed using SAS program (SAS Institute 1989). The mean differences were analyzed by Student-Newman-Keuls Test at the *P* < 0.05 significance level.

## 3. Results and Discussion

### 3.1. Effect of Mercury on Seedlings Growth, Biomass and Relative Water Content

**Figure 1:**
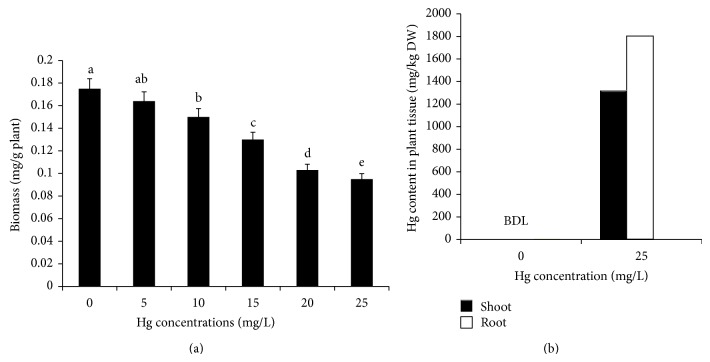
Biomass (a) and mercury accumulation (b) level in* M. arvensis *seedlings after 12 days of mercury treatment along with untreated control. Values are means (*n* = 3) ± SE; bars followed by the same letters are not significantly different for *P* ≤ 0.05 according to the Duncan's test; BDL: below detectable limit.

Shoot and root growth was varied depending on the concentrations of Hg treatment. After 12-day treatment, the seedling growth was decreased to 56.1% and 41.5% in root and shoot tissues, respectively at 25 mg/L Hg dose compared to the control (Table 1). The effect of Hg treatment on seedling biomass was presented in Figure 1(a). The seedling biomass was gradually decreased with increasing the Hg dose level in the growth medium. The maximum biomass reduction noticed was 45.7% in Hg treated seedlings (25 mg/L Hg dose) compared to the control. Both seedlings growth and overall biomass were found to be decreased by Hg exposure. The growth reduction observed in plants that were subjected to heavy metal concentration often results from direct effects (toxicity of heavy metals accumulated in tissue) or from indirect effects (limitation of mineral and water acquisition). The inhibition was stronger in root tissues than shoot at higher Hg concentration. When uptake of nutrition was inhibited in roots, the growth of whole plants was constrained and the plant biomass was decreased ultimately [32, 33]. The reason is that plant roots were the first point of contact with these toxic mercury ions in the growth medium. A similar result was also reported by Zhou et al. [34]. Plant biomass is a good indicator for characterizing the growth performance of heavy metal stressed plants.* M. arvensis* seedling biomass was decreased with increasing of Hg concentrations in growth medium (Figure 1(a)). The present result is in agreement with earlier report by Cavallini et al. [35]. The relative water content was slightly changed due to Hg treatment (Table 1).

### 3.2. Accumulation of Mercury in* M. arvensis* Seedlings

The result related to the bioaccumulation of Hg content in* M. arvensis* was depicted in Figure 1(b). The maximum level of Hg accumulation noticed was 1816.54 mg/kg DW and 1331.50 mg/kg DW for root and shoot tissues, respectively, at 25 mg/L Hg exposure compared to the control. The level of Hg accumulation was found to be high in root than in shoot tissues. Hence, the translocation of Hg ions from root to shoot tissues was found to be low. Accumulation of higher level of Hg content in root system suggests that roots serve as a partial barrier for the transport of mercury to shoots [36]. Similar result was also reported by Singh et al. [37].

### 3.3. Effect of Mercury Exposure on Chlorophyll Pigment Contents

The data on total chlorophyll (Chl *a*, Chl *b*) and caroteniod contents of* M. arvensis* seedlings exposed to differing concentrations of Hg were illustrated in Table 2. The level of chlorophyll pigment contents was decreased with increasing the Hg concentrations compared to the control (Table 2). The percentage of chlorophyll pigment contents inhibition noticed was 29.3%, 24.3%, and 29.0% for Chl *a*, Chl *b*, and carotenoid, respectively, at 25 mg/L Hg treatment. The decreased level of photosynthetic pigments may be attributed due to the Hg induced inhibition of chlorophyll and carotenoid biosynthesis possibly caused by nutrient deficiency, such as Mn, Cu, Fe, and P [38, 39]. Similar results were also reported in* Medicago sativa *under the Hg stress [34].

### 3.4. Effect of Mercury Induced Stress on Antioxidative Enzyme Activities and Total Soluble Protein Contents

**Figure 2:**
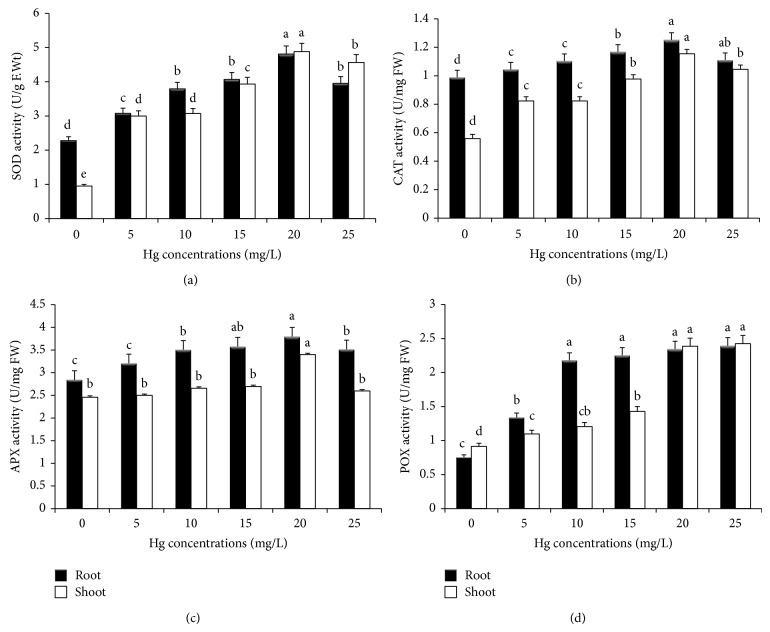
Effect of mercury heavy metal induced stress on SOD (a), CAT (b), APX (c), and POX (d) activities of* M. arvensis *seedlings after 12 days of treatment along with untreated control. Values are means (*n* = 3) ± SE; bars followed by the same letters are not significantly different for *P* ≤ 0.05 according to the Duncan's test.

Heavy metals induce oxidative stress by generation of superoxide radical (O_2_^−^), hydrogen peroxide (H_2_O_2_), hydroxyl radical (HO^•^), and singlet oxygen (^1^O_2_) that are collectively termed as reactive oxygen species (ROS) [40, 41]. ROS can rapidly affect various biomolecules such as nucleic acid, proteins, lipids, and amino acids [42]. Therefore, the enhancement of various antioxidant enzymes level (SOD, CAT, APX, and POX) is an important protective mechanism to minimize the oxidative damage occurring in the stressed plants. SOD plays a key role in cellular defense mechanisms against reactive oxygen species (ROS). The effect of Hg exposure on SOD activity was presented in Figure 2(a). The SOD activity was linearly increased with increasing the Hg concentrations in both root and leaf tissues. The maximum percentage of SOD activity observed for root and leaf tissues was 211.1 and 527.7, respectively, at 20 mg/L Hg treatment. An increase in SOD activity may be linked to an increase in superoxide radical formation as well as to the de novo synthesis of enzyme protein [43]. The present result indicated that the increase of SOD activity at lower dose of Hg treatment might protect* M. arvensis* seedlings from the oxidative injury. However, the SOD activity was slightly decreased at 25 mg/L Hg treatment. The decrease of SOD activity at higher concentration of Hg treatment might be attributed to enzyme damage due to the excessive production of free radicals and peroxides.

CAT is one of the most efficient antioxidant enzymes and it plays an important role in maintaining the redox homeostasis of the cell [44]. There was increasing trend in CAT activity with Hg treatment; however, it was slightly declined at higher dose (Figure 2(b)). Maximum CAT activity recorded was 112.2% and 276.3% for root and leaf tissues reported at 20 mg/L Hg treatment. This result suggested that* M. arvensis *has a great ability to cope with oxidative stress caused by Hg. Cho and Park [45] reported that CAT activity increased gradually with increasing of Hg concentrations in* Jatropha curcas* plants exposed to Hg.

APX has high affinity to detoxify the H_2_O_2_ and it reduces H_2_O_2_ into water using ascorbate as the electron donor, resulting in the formation of dehydroascorbate. The APX activity was also enhanced at lower concentrations of Hg treatment, but it was slightly inhibited when the concentration was increased beyond 20 mg/L Hg treatment (Figure 2(c)). The maximum APX activity increase noticed was 138.4% and 102.17% for roots and leaves, respectively, at 20 mg/L Hg exposure, compared to the control, which indicated that possible mechanism has evolved in* M. arvensis* seedlings against the Hg induced oxidative stress. Similar results were also noticed in* Phaseolus aureus* [46] and* Alfalfa *[38].

Peroxidase is widely distributed in the plant kingdom and is one of the principal enzymes involved in elimination of active oxygen species (AOS). The effect of Hg exposure on POX activity was illustrated in Figure 2(d). POX activity showed an increasing trend with increasing Hg concentrations compared to the control. Maximum POX activity observed was 317.6 % and 245.2% for root and leaf tissues, respectively, at 25 mg/L Hg dose. This result indicated that* M. arvensis* has the effective mechanism to detoxify the oxidative damage caused by Hg stress. Among the four antioxidative enzymes, POX activity was found to be higher in both roots and leaves and it is considered as the stress marker antioxidative enzyme. Increased POX activity has been previously reported in* Alfalfa *[38], Tomato [47], and Cucumber [48] plants that were exposed to mercury stress. In the present study, antioxidative enzymes such as SOD, CAT, APX, and POX activity were found to be higher in root than in leaf tissues of* M. arvensis* under Hg exposure. The increased SOD, CAT, APX, and POX activities in* M. arvensis* may be considered as circumstantial evidence for Hg heavy metal tolerance mechanisms developed by this plant species.

**Figure 3:**
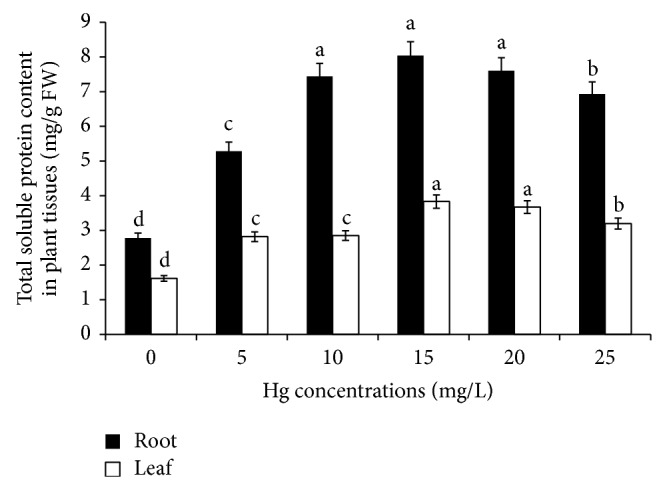
Effect of mercury induced stress on total soluble protein content in* M. arvensis *seedlings after 12 days of treatment along with untreated control. Values are means (*n* = 3) ± SE; bars followed by the same letters are not significantly different for *P* ≤ 0.05 according to the Duncan's test.

The protein content may be considered a reliable indicator of oxidative metal stress in plants [38]. The effect of Hg treatment on total soluble protein content was represented in Figure 3. Total soluble protein content was increased up to 15 mg/L Hg treatment in roots and leaves compared to control. The maximum level of total soluble protein content observed was 32% and 42% for root and leaf tissues, respectively, at 15 mg/L Hg exposure. The protein content was slightly decreased at higher dose of Hg treatment. This increase may be due to the increasing activity of some other metal sequestration mechanisms, involved in the detoxification of high heavy metal doses [49]. However, the total soluble protein content was decreased at higher concentrations of Hg exposure. It seems that due to high Hg content accumulation in root and leaf tissues, these might have greater generation of ROS and, hence, more oxidative stress that might have resulted in decreased level of protein content through oxidative damage. Cargenelutti et al. [47] reported that the soluble protein content was increased at 250 *μ*mol Hg dose and it was decreased at higher Hg concentration in cucumber seedlings. It is interesting to note that all the enzyme activities and total soluble content were found to be higher in root than in leaf tissues due to the Hg heavy metal treatment.

### 3.5. Effect of Mercury Ion Stress on RAPD Banding Pattern

**Figure 4:**
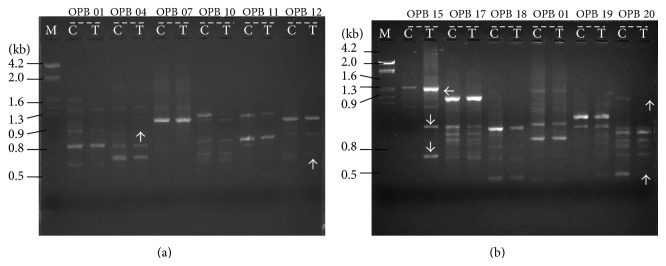
RAPD profiles of genomic DNA isolated from the leaves of* Mentha arvensis* seedlings after 12 days of Hg treatment along with control. Lane M: molecular marker; Lane C: control; Lane T: 25 mg/L Hg treatment; ↑: disappearance of normal bands; ↓: appearance of new bands; →: intensity of bands.

In the present study, a total of 40 random oligonucleotide primers were tested and 12 primers were selected for developed of stable RAPD banding pattern. The details of all polymorphic and monomorphic bands in RAPD profile were presented in Table 3 (Figures 4(a) and 4(b)). RAPD profiles showed significant differences between control and Hg treated samples. The principal observation or changes in the RAPD profiles included the variation in band intensity, disappearance of bands, and appearance of new bands compared with the control plants. The molecular size of the two additional DNA bands obtained with OPA 15 primer was 800 bp and 900 bp in 25 mg/L Hg treatment. Further the DNA band intensity was increased at 25 mg/L Hg dose compared to the control. In the case of OPA 19 primer, a 1500 bp DNA band was amplified from 25 mg/L Hg treated leaf DNA sample and this band did not appear in control DNA sample. The leaf DNA amplification with OPA 20 primer revealed that two DNA fragments (500 bp and 1300 bp) were disappeared at 20 mg/L Hg dose. With the primer OPB 12, 800 bp DNA amplicon was absent at 25 mg/L Hg exposure. However, the DNA band intensity was decreased at 25 mg/L Hg treatment compared to the control. The RAPD patterns of primer OPB 04, a 1400 bp band was not amplified in 25 mg/L Hg treatment. The genomic template stability (GTS) was calculated for each primer and presented in Table 3. The disappearance of normal RAPD bands may be related to the events such as DNA damage and point mutations that are induced by genotoxins [49]. Chen et al. [22] reported similar type DNA damage induced by Cd heavy metal treatment increase and/or decrease of band intensity, disappearance of normal bands, and appearance of new bands in barley seedlings. The present results strongly suggested that Hg heavy metal treatment induced DNA changes at genome level in* M. arvensis*. The appearance of new bands might be responsible for hyperaccumulation of Hg metal ions. It is suggested that appearances of new bands may be attributed to mutations, while the disappearances of normal bands may be associated with DNA damage. A comparable analysis of molecular and physiochemical future may illustrate several advantages. For instance, a typical reduction of* Mentha* seedlings growth doses associated with a significant inhibition in DNA replication induces that the occurrence of DNA damage may be essential in the majority of the cells. In the presence study, it is likely that, for Hg doses used, DNA replication was decreased due to the increased level of DNA damage. It is noteworthy to mention that as seedlings growth and photosynthetic pigments content in range of 5–25 mg/L Hg treatment showed a negative correlation compared to the control, while, biochemical parameters in range of 5–25 mg/L Hg treatment displayed a positive correlation than the control, this could be assumed that the DNA damages were efficiency repaired to certain extent by* Mentha *seedlings. This is one of the reasons why the seedlings efficiently survived at 25 mg/L Hg treatment and hyperaccumulation Hg ion content in the seedling tissues.

## 4. Conclusions

In conclusion, accumulation of Hg heavy metal ions in plant tissues induced both physiochemical and molecular changes in* M. arvensis* seedlings. The data confirm that the occurrence of phytotoxic effect of Hg treatment was observed at the higher dose. Thus the* M. arvensis* plant has efficient detoxification potential to scavenge excess of ROS very efficiently by activation of SOD, CAT, APX, and POX antioxidative defense system together in a coordinated way. Further the increase of total soluble protein content indicated that* M. arvensis* plants have the ability of the detoxification of heavy metal ions by triggering the antioxidative defense systems under Hg induced stress. The present results showed that the occurrence of changes in RAPD patterns including DNA band intensity, absence, and presence of additional DNA bands in* M. arvensis* seedlings might be due to the Hg heavy metal induced genotoxicity. To the best of our knowledge, this is the first report describing the effect of Hg heavy metal stress induced physiochemical and molecular changes in* M. arvensis*. Because of the ability to grow and tolerate mercury toxicity,* Mentha arvensis* could be considered as a promising plant species for phytoremediation of heavy metals.

## Figures and Tables

**Table 1:** Effect of mercury heavy metal exposure on seedlings growth, biomass, and relative water content of *M. arvensis*.

Hg conc. (mg/L)	Shoot length (cm)	Root length (cm)	IT values (%)		RWC (%)
			Shoot	Root	
0.0	26.50 ± 1.55^*^	22.25 ± 1.37^*^			86.6
5.0	19.25 ± 0.85^a^	13.00 ± 0.91^a^	72	58	86.3
10.0	19.00 ± 1.49^a^	12.15 ± 1.29^a^	70	53	87.0
15.0	18.75 ± 1.87^a^	12.00 ± 1.27^a^	71	55	86.0
20.0	17.50 ± 1.04^b^	10.75 ± 0.85^b^	66	50	87.5
25.0	15.50 ± 1.32^c^	9.75 ± 0.85^b^	58	43	87.0

^*^Data are means ± SE (*n* = 3); columns with different letters indicate significant differences at P ≤ 0.05.

**Table 2:** Effects of mercury heavy metal induced stress on chlorophyll a, b and carotenoid contents in leaves of *M. arvensis* seedlings along with untreated control.

Hg Concen. (mg/L)	Photosynthetic pigments (mg/g fw)			
	Chl a content	Chl b content	Total Chl content	Car content
0.00	1.574 ± 0.001^a^	0.584 ± 0.009^a^	2.158 ± 0.010^a^	0.539 ± 0.001^a∗^
5.0	1.561 ± 0.001^a^	0.571 ± 0.014^a^	2.132 ± 0.015^a^	0.537 ± 0.001^a^
10.0	1.530 ± 0.006^a^	0.530 ± 0.008^a^	2.060 ± 0.014^b^	0.536 ± 0.001^a^
15.0	1.431 ± 0.135^b^	0.462 ± 0.074^b^	1.893 ± 0.209^c^	0.436 ± 0.002^b^
20.0	1.195 ± 0.008^c^	0.456 ± 0.009^b^	1.651 ± 0.017^d^	0.438 ± 0.002^b^
25.0	1.112 ± 0.018^d^	0.442 ± 0.011^b^	1.554 ± 0.029^e^	0.426 ± 0.004^b^

^*^Data are means ± SE (*n* = 3); columns with different letters indicate significant differences at *P* ≤ 0.05.

**Table 3:** List of RAPD primers, their sequences, GC content, changes of total bands, and genomic template stability (GTS %) in control and Hg treated leaves of *M. arvensis* seedlings.

Number of primer	Name of primers	Sequences 5′ to 3′	GC content (%)	Total number of bands in control and Hg treated laves					
				Control	25 mg/L Hg				GTS (%)
					*a*	*b*	*C*	*d*	
Primer 1	OPB-01	GTT TCG CTC C	60	0	2	0	1	0	—
Primer 2	OPB-04	GGA CTG GAG T	60	7	1	0	1	3	—
Primer 3	OPB-07	GGT GAC GCA G	70	5	0	2	0	1	39.0
Primer 4	OPB-10	CTG CTG GGA C	70	6	1	3	0	2	49.0
Primer 5	OPB-11	GTA GAC CCG T	60	2	4	0	0	1	—
Primer 6	OPB-12	CCT TGA CGC A	60	8	0	3	2	0	36.5
Primer 7	OPB-15	TCC GCT CTG G	70	7	0	3	1	2	41.0
Primer 8	OPB-17	AGG GAA CGA G	60	7	1	2	0	1	27.0
Primer 9	OPB-18	CCA CAG CAG T	60	4	0	0	0	0	—
Primer 10	OPA-01	CAG GCC CTT C	70	6	0	1	0	1	15.6
Primer 11	OPA-19	CAA ACG TCG G	60	3	0	1	0	1	32.3
Primer 12	OPA-20	GTT GCG ATC C	60	5	0	3	0	0	59.0
